# Prediction of protein-protein binding site by using core interface residue and support vector machine

**DOI:** 10.1186/1471-2105-9-553

**Published:** 2008-12-22

**Authors:** Nan Li, Zhonghua Sun, Fan Jiang

**Affiliations:** 1 Beijing National Laboratory for Condensed Matter Physics, Institute of Physics, Chinese Academy of Sciences, Beijing 100080, PR China; 2 Graduate School of the Chinese Academy of Sciences, PR China

## Abstract

**Background:**

The prediction of protein-protein binding site can provide structural annotation to the protein interaction data from proteomics studies. This is very important for the biological application of the protein interaction data that is increasing rapidly. Moreover, methods for predicting protein interaction sites can also provide crucial information for improving the speed and accuracy of protein docking methods.

**Results:**

In this work, we describe a binding site prediction method by designing a new residue neighbour profile and by selecting only the core-interface residues for SVM training. The residue neighbour profile includes both the sequential and the spatial neighbour residues of an interface residue, which is a more complete description of the physical and chemical characteristics surrounding the interface residue. The concept of core interface is applied in selecting the interface residues for training the SVM models, which is shown to result in better discrimination between the core interface and other residues.

The best SVM model trained was tested on a test set of 50 randomly selected proteins. The sensitivity, specificity, and MCC for the prediction of the core interface residues were 60.6%, 53.4%, and 0.243, respectively. Our prediction results on this test set were compared with other three binding site prediction methods and found to perform better. Furthermore, our method was tested on the 101 unbound proteins from the protein-protein interaction benchmark v2.0. The sensitivity, specificity, and MCC of this test were 57.5%, 32.5%, and 0.168, respectively.

**Conclusion:**

By improving both the descriptions of the interface residues and their surrounding environment and the training strategy, better SVM models were obtained and shown to outperform previous methods. Our tests on the unbound protein structures suggest further improvement is possible.

## Background

The functions of proteins rely on their interactions with various biological molecules including proteins, DNAs, RNAs and other small molecules. Among those interactions, one of the most important ones is the protein-protein interaction. Hence, the identification of protein binding site for protein-protein interaction becomes one of the basic questions in the research of protein functions. Several experimental methods such as X-ray crystallography, NMR, and site-directed mutagenesis [1] are well established in providing structural information on the protein-protein binding site. But the proteomics research is currently generating tremendous protein interaction data [2] in want of detailed annotation by structural information. According to the current capability of experimental methods for obtaining structural information, only a limited amount of the proteomics-generated data can be processed and annotated [3]. Therefore, the computational prediction methods such protein binding site prediction have become very important alternatives to interpret and annotate the experimentally generated proteomics data.

The computational prediction of protein binding site is particularly helpful in improving the speed and accuracy of protein docking method [4,5]. A protein docking method predicts the structure of a protein-protein complex from the structures of its monomers and can provide detailed structural information for protein-protein interactions. Docking methods can be usually divided into two parts: sampling of complex conformations given the structures of the monomers and scoring of these conformations in order to find the near-native conformations. If the information of the binding site could be known in advance, the speed and accuracy of the docking method could be significantly improved, because the process of conformation sampling could be restricted to a relative small area close to the binding site. Some of the recent studies [6,7] have applied the information of predicted binding site residues to the process of docking.

The binding site prediction method is mainly based on the following hypothesis. First, the characteristics of interface and non-interface residues are significantly different. Second, these differences can be quantified and utilized to design methods to discriminate and hence predict the binding sites.

The characteristics of the binding sites, namely the interface residues, have been systematically studied [8-14]. Several previous works have found that the amino acid composition is different between the interface and the non-interface residues. Lo Conte et al. [8] have analyzed the amino acid composition on different parts of protein-protein complexes using a dataset of 75 protein complexes. They found that the interface residues contain more aromatic and aliphatic residues than the non-interface residues. They concluded that the amino acid composition of the interface residues is more similar to that of the interior residues than to that of the non-interface residues. Neuvirth et al. [9] also found that some polar and aromatic residues are more abundant in the interface than outside the interface, which is similar to Lo Conte's conclusion. They also found that hydrophobic residues tend to clusters on the interface.

Furthermore, some studies found that the interface residues are more conserved than the non-interface residues. Zhou and Shan [10] found that the sequence conservation works well for the discrimination of interface residues from non-interface residues in their site-prediction methods. In their latest work [15], they compared the conservation scores of interface and surface residues and showed that interface residues are more conserved. Hu et al. [16] and Ma et al. [17] analyzed the residue conservation in several protein families and found that the polar residues are highly conserved in the interface.

The secondary structure composition of interface residues was also studied by several researchers. Jones and Thornton [18] found that interface residues prefer to be helix or coil rather than sheet. However, Neuvirth et al. [9] found that the secondary structures of interface residues prefer to be sheet or coil rather than helix. They explained the contradictory results by considering the differences in the database analyzed. So there exists a variety in the composition of secondary structures on the interface.

Lo Conte et al. [8] and Chakrabarti et al. [11] analyzed the shape of the interface region and defined the interface atoms into two classes. The first class of atoms locates in the core region of the interface and the second class surrounds the first class and locates on the rim of the interface.

Based on the studies on the characteristics of interface, several methods have been developed to predict and identify the interface residues from all residues on the protein surface [9,10,15,19-38]. Various features have been used to describe the characteristics of the interface. Most of them combine several properties of amino acid residues together. Some of the common features that have been used are sequence conservation [9,10,20-27], accessible surface area [10,28-30], and amino acid composition [20,29,30]. The frequently used algorithms to identify interface residues from all surface residues are evolutionary tracing [21-25], probability estimation [9], linear parameter optimization [20], neural network [19,31], and support vector machine learning [30,32-34].

Neuvirth et al. [9] designed ProMate and applied nine different properties to describe the characteristics of a surface patch. A probability estimation method was used to estimate the probability of the patch to be a part of the interface according to the values of nine properties. Liang et al. [20] designed PINUP and the central (or interaction) residue is described by the combination of side chain energy score, residue conservation score and residue interface propensity. The three properties were linearly combined into one score and the weights were obtained by a linear parameter optimization method. Chen et al. [19] and Tjiong et al. [15] designed the cons-PPISP method which used the sequence profile of the central residue and relative accessible surface area to describe the residues. A consensus neural network method was used to separate the interface residues from the non-interface residues. SVM is one of the most frequently used machine learning methods applied to the prediction of interface residues [30,32,33]. Yan et al. [32] used a combination of SVM and Bayesian network with a sequence profile of the central residue and its sequence neighbours to make interface prediction. Koike et al. [33] used SVM with a profile of sequence and space neighbours of the central residue. Bradford et al. [30] used a patch description of protein surface and the prediction of interface patches was performed by SVM.

In this paper, we designed an interface residue prediction method based on SVM by using the concept of core interface residue and by designing several new properties for the description of both sequentially and spatially neighbouring residues. It was found that the core interface residues were more effective in training SVM models. The training and testing were performed using structures taken from a database of the complex structures from the PDB [39]. The prediction results of our method outperformed several other prediction methods such as ProMate, PINUP, and cons-PPISP. The unbound proteins from the protein-protein interaction benchmark [40] were also used to test our method. The results showed that our method could make reasonable prediction for the unbound structures as well.

## Results

### Statistics on the amino acid composition of the neighbour residues

**Figure 1:**
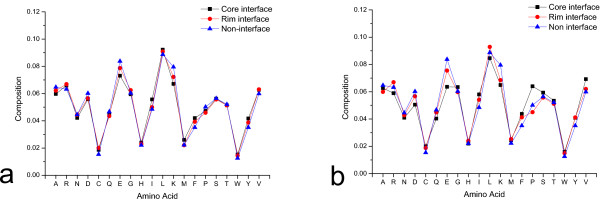
**Comparison of the amino acid compositions of the neighbour residues for the three residue classes**. In this figure, the amino acid compositions of the neighbour residues for the core interface, the rim interface, and the non-interface residues are compared. Colour black, red, and blue represent the core interface, the rim interface and the non-interface residues, respectively. a) Core cut-off equals to 0.2. b) Core cut-off equals to 0.8.

The amino acid composition of the sequence and space neighbour residues of the central residues were calculated. Then, the average compositions were calculated for the three residue classes respectively. The three classes are core interface, rim interface and non-interface, which are all surface exposed residues. The results are shown in Figure 1. (See Additional file 1 for the p-values of the Welch t-test for all the residue composition data of core interface, rim interface, and non-interface residues).

Figure 1a shows the result using core cut-off = 0.2. Amino acid residues I, M, F, Y, and V appear more frequently in the neighbours of the core interface residues than in either of the other two residue classes or only the non-interface class. This indicates that the core interface residues are more likely to be hydrophobic. D, E, and K appear more frequently in the neighbours of the non-interface residues than in that of the other two residue classes. This indicates that the non-interface residues are more likely to be polar. The compositions of the rim interface residues indicate that for some amino acids, the rim interface resembles the core interface, while for others, it resembles the non-interface.

**Figure 2:**
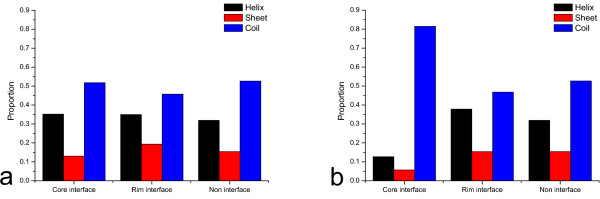
**The secondary structure compositions of the three residue classes**. In this figure, the secondary structure compositions of the core interface, the rim interface, and the non-interface residue are compared. The bars in black, red, and blue represent the percentage of helix, sheet, and coil in the three residue classes, respectively. a) Core cut-off equals to 0.2. b) Core cut-off equals to 0.8.

**Figure 3:**
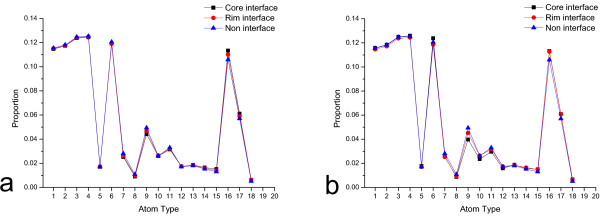
**Comparison of the atom compositions of the neighbour residues for the three residue classes**. In this figure, the atom compositions of the neighbour residues for the core interface, the rim interface, and the non-interface residues are compared. Colour black, red, and blue represent the core interface, the rim interface and the non-interface residues, respectively. The details of the 18 atom types can be found in the Additional file 2. a) Core cut-off equals to 0.2. b) Core cut-off equals to 0.8.

When further observation on core residues is compared using a higher core cut-off of 0.8 (Figure 1b), it is seen that in addition to the similar trend for core cut-off 0.2, G and P become more preferable to be the neighbour of the core residues than other two residue classes. It is probably due to the preference of coil state for the core interface residue. On the other hand, R becomes less preferable to be the neighbour of the core interface residues than other two residue classes. For the neighbours of rim interface, L and R become more preferable and P becomes less preferable. It is probably due to their innate secondary structure propensities as the rim interface residues have distinct preference for helix and sheet state as shown in Figure 2. Besides P, A also becomes less preferable to be the neighbour of rim interface. It is consistent with the result (Figure 3b) that beta carbon atoms are less preferable to be the neighbour of the rim interface. The data for the composition of rim interface indicates that the rim interface probably does not like residues with short side-chains and prefers residues with long side-chains.

When the p-value for significant differences is set as 0.05, for both core cut-offs, all residues except G, S and T show significant difference in the average composition between the core interface and the non-interface. Residues A, C, D, E, F, G, K, P, R, W, and V show significant difference in the average composition between the rim interface and the non-interface. Residues A, E, F, G, I, K, M, N, and Y show significant difference in the average composition between the core interface and the rim interface. For core cut-off 0.2, residue C shows significant difference in the average composition between the core interface and the rim interface. Residue T shows significant difference in the average composition between the rim interface and the non-interface. For core cut-off 0.8, residue G shows significant difference in the average composition between the core interface and the non-interface. Residues M and Y shows significant difference in the average composition between the rim interface and the non-interface.

### Statistics on the atom composition of the neighbour residues

The atom compositions of the sequence and space neighbours of the central residues were calculated for each central residue. Then, the average compositions for each atom type were calculated for the three residue classes respectively and shown in Figure 3 (see the Method section and Additional file 2 for the details of the 18 atom classes [41] and Additional file 3 for the p-values of the Welch t-test between some of the atom composition data of core interface, rim interface, and non-interface). Among 18 atom types, five show clear difference in the atom composition for both core cut-offs and only two atom types for core cut-off 0.8.

Atom types 16 and 17 are preferred to be in the neighbour of the core interface residues. These two atom types mainly contain the gamma or delta carbon atoms of the side chains of several hydrophobic and aromatic residues. This preference indicates that the core interface is generally hydrophobic. Atom types 7, 8, and 9 are preferred to be in the neighbours of the non-interface residues. These three atom types mainly contain nitrogen, oxygen, and carbon atoms of several charged residues. This preference of atom types 7, 8, and 9 indicates that the non-interface is generally polar.

For the core residues classified by the cut-off 0.8 (Figure 3b), atom class 6, CB of all residues and most carbon atoms of Pro, preferred to be in the neighbour of the core interface residues. Atom type 10 and 11 preferred to be in the neighbour of non-interface residues. These two atom types contain nitrogen, oxygen, and some of the carbon atoms of several charged and polar residues. This preference of atom type 10 and 11 also indicates that the non-interface is generally polar.

### Statistics on the secondary structure composition of the central residues

The secondary structure compositions of the core interface, the rim interface, and the non-interface residues are shown in Figure 2. When the core cut-off is 0.2, the statistics on the secondary structures (Figure 2a) for all three classes of surface residues show a similar pattern, that is, helix and coil are more abundant than sheet. When the core cut-off is increased to 0.8, although the rim and non-interface still have the similar pattern, a clear difference is seen for the core interface (Figure 2b). The core interface is strongly preferred to be in the coil state. This pattern of secondary structure preference suggests that the core interface may be more flexible than the rim and non-interface regions.

### Statistics on the side-chain environment

The side-chain environment compositions of the core interface, the rim interface, and the non-interface residues are shown in Table 1. The definition of side-chain environment can be found in the Method section. Whether the core cut-off is 0.2 or 0.8, the composition of side-chain environment is biased on environment states E and P_1_, because most of the residues in the three interface classes are surface exposed and thus belong to either E (exposed) or P (partial buried) states. The majority of them are in E, a fifth of them in P_1_, and the rest of four environment states occupy about 10%. Comparing statistics of core cut-offs at 0.2 and 0.8, it can be seen that the core interface are more exposed as more stringent criterion is applied to define this class, that is, using a higher core cut-off. This is consistent with the above result of seeing more core interface residues in the coil state.

**Table 1:** Composition of side-chain environment class

**Core cut-off**	**Residue class**	**E**	**P**_1_	**P**_2_	**B**_1_	**B**_2_	**B**_3_
0.2	Core interface	75.2%	18.7%	3.8%	1.9%	0%	0.3%
	Rim interface	53.2%	30.1%	8.2%	7.8%	0.1%	0.6%
	Non-interface	65.5%	22.0%	5.6%	4.9%	0.1%	0.5%

0.8	Core interface	93.4%	5.4%	0.6%	0%	0%	0%
	Rim interface	67.9%	22.9%	5.2%	3.5%	0%	0.4%
	Non-interface	65.5%	23.5%	5.6%	4.9%	0.1%	0.5%

This table shows the composition of residues in each side-chain environment class. The first column is the value of core cut-off. The second column is the name of the three residue class. The third column to the eighth column shows the residue composition of each environment class for each residue class. The sum of the compositions of six classes does not equal to 1 because some of the residues cannot be classified into either of the class due to the loss of accessible surface area data. The classification method of E, P_1_, P_2_, B_1_, B_2_, and B_3 _is shown in the Method section.

### Training of SVM models for different core cut-offs

As mentioned in the Method section, the residues of the 927 proteins (see Additional file 4 for the list of the 927 proteins of the training set) could be divided into four classes: the interior residues, the core interface residues, the rim interface residues, and the non-interface residues. The numbers of residues for each class of different core cut-offs are shown in Table 2.

**Table 2:** Residue numbers for each residue class

**Core cut-off**	Core interface residue	Rim interface residue	Non-interface residue	Interior residue
0.2	38264	9803	70147	74453
0.5	19042	29025	70147	74453
0.8	5149	42918	70147	74453

This table shows how many residues there are in each residue class. The first column is the core cut-off for different classification. The second to the fifth columns are the number of residues for the core interface, the rim interface, the non-interface, and the interior residues respectively.

The training set for SVM consisted of 10000 random selected core interface residues and 10000 residues which were randomly selected from the rim-interface and non-interface residues. The training set for the core cut-off of 0.8 consists of 5149 core interface residues and the same number of residues which were randomly selected from the rim-interface and non-interface residues.

**Figure 4:**
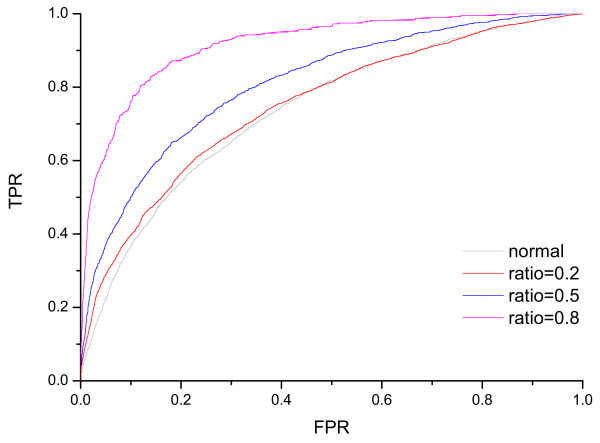
**The ROC curves for different models**. In this figure, the ROC curves for different SVM models are presented. The gray curve is generated using models to discriminate interface from non-interface residues. The red, blue, and pink curves are generated using models to discriminate core interface from other residues. The core cut-offs for red, blue, and pink curves are 0.2, 0.5, and 0.8 respectively. The AUC for the gray, red, blue, and pink curves are 0.7385, 0.7498, 0.8184, and 0.9169 respectively.

The CVA (Table 3) and AUC of ROC curves (Figure 4) are both compared to illustrate the discrimination performance of different models when the core cut-off varies. The discrimination ability of the respective SVM model increases as the core cut-off increases. The best model is generated when the core cut-off equals to 0.8, the CVA is 84.8% and the AUC is 0.9169. Moreover, all the models that discriminate core interface residues against other residues are better than the 'normal' model that discriminate the interface residues from the non-interface residues defined by an interface contact distance of 5 Angstrom.

**Table 3:** Cross validation accuracy for different models

**Model**	**Cross Validation Accuracy**
'Normal'	68.2%
Core cut-off = 0.2	69.2%
Core cut-off = 0.5	74.8%
Core cut-off = 0.8	84.8%

'Normal' stands for the model trained by interface and non-interface residues without using the definition of core interface residues. Core cut-off = X (0.2, 0.5, 0.8) corresponds to the model trained by core interface, rim and interface residues. The second column is the 5-fold cross validation accuracy.

### Contribution of each descriptor from leave-one-out tests

Eight residue descriptors and four central-residue specific descriptors were described in the Method section. To verify their effects in the prediction of binding site, descriptor selections based on the leave-one-out test were performed (Table 4). In the leave-one-out test, descriptors were removed one at a time. The values of CVA were compared between the results of using all descriptors and that with the selected descriptor removed. The leave-one-out tests were performed for the training sets of core cut-offs equal to 0.2 and 0.8.

**Table 4:** Contribution of each descriptor

**Composition of feature vector**	**Core cut-off = 0.2**	**Core cut-off = 0.8**
All descriptors	69.2%	84.8%
Without number of atom	0%	-0.1%
Without number of charge	-0.2%	-0.2%
Without number of H-bond	-0.1%	0%
Without hydrophobic index	-0.3%	-0.1%
Without relative accessible surface area	-0.6%	-0.8%
Without secondary structure	-0.2%	-0.3%
Without conservation score	-1.4%	-0.9%
Without side-chain environment	-0.4%	-0.5%
Without sequence distance	-0.1%	-0.1%
Without spatial distance	-0.2%	0%
Without residue composition	-0.4%	-0.1%
Without atom composition	-0.1%	-0.2%
Without total charge number	-0.1%	0%
Without total H-bond number	-0.1%	0%

This table shows the results of leave-one-out test for models built when the core cut-offs equal to 0.2 and 0.8 respectively. The first column is the composition of feature vectors. The second and the third columns are the CVA results of core cut-off = 0.2 and core cut-off = 0.8, respectively. The second line shows the CVA results of using all descriptors of both cut-offs. The values from the third line to the sixteenth line are the difference between the CVA of each leave-one-out test and the CVA using all descriptors.

The change of CVA for each descriptor was shown in Table 4. Conservation is the most important factors for both core cut-off = 0.2 and core cut-off = 0.8. After the removal of the conservation, the CVA decreased by 1.4% and 0.9% for core cut-off = 0.2 and core cut-off = 0.8 respectively (Table 4). Relative accessible surface area and local environment are the second and the third most important descriptors. The decreases of CVA after removing relative accessible surface area are 0.6% and 0.8% for core cut-off = 0.2 and core cut-off = 0.8 respectively. The decreases of CVA for local environment are 0.4% and 0.5% for core cut-off = 0.2 and core cut-off = 0.8 respectively. The decrease of CVA after removing all other descriptors vary from 0.1%–0.4% for core cut-off = 0.2 and 0.1%–0.3% for core cut-off = 0.8. Therefore, all these descriptors show positive effects in the discrimination between the core residues and other residues.

### Prediction results on a test set and comparison with other methods

A test set containing 50 proteins (therefore 50 distinct binding interfaces/sites) was used (see Additional file 5 for the list of 50 proteins of the test set). Feature vectors were generated for each surface residue on the proteins. The prediction was performed as mentioned in the Method section. Then, the sensitivity, specificity, and MCC were calculated for each protein chain. The average sensitivity, specificity, and MCC of the prediction for the test set were 60.6%, 53.4%, and 0.243, respectively (Table 5) for the model using core cut-off 0.2. The average sensitivity, specificity, and MCC of the prediction for the test set were 60.2%, 18.2%, and 0.236, respectively (Table 5) for the model using core cut-off 0.8. The deterioration of specificity of the model using core cut-off 0.8 is mainly because the biased number between the core interface and other residues. Therefore, we applied the model of core cut-off 0.2 to perform the comparison tests between other methods.

**Table 5:** Prediction results on test set using different core cut-offs

**Core cut-off**	**Sensitivity**	**Specificity**	**MCC**
0.2	60.6%	53.4%	0.243
0.8	60.2%	18.2%	0.236

This table shows the prediction results on test sets by models using different core cut-offs. The first column is the core cut-off used to make classification. The second to the fourth columns are the average sensitivity, specificity, and MCC for the 50 proteins in the test set respectively.

It was generally difficult to compare the results of different methods because of the different definition of interface residues and evaluation methods. We performed tests using the above test set on several different binding site prediction methods, including ProMate [9], PINUP [20], and PPISP [15,19]. All predictions were performed via the internet on their web servers. Data were first submitted to the servers and then analysis was performed on the prediction results received from the servers. The definition of interface and non-interface residues followed the description in the Method section.

The prediction results on all the servers are shown on Table 6. The sensitivities for ProMate, PINUP, and PPISP were 9.9%, 21.2%, and 27.7% respectively. The specificities for ProMate, PINUP, and PPISP were 28.1%, 39.5%, and 44.2%, respectively. The MCCs for ProMate, PINUP, and PPISP were 0.009, 0.096, and 0.146, respectively. For comparison, we directly did the statistics on the interface residues instead of on the core interface residues in the prediction result of our method. The sensitivity, specificity, and MCC of our prediction result using the model of core cut-off = 0.2 are 60.7%, 41.9%, and 0.203 respectively. From the above comparisons, it can be seen that our prediction method is generally more accurate than the other three methods.

**Table 6:** Prediction results on test set

**Prediction method**	**Sensitivity**	**Specificity**	**MCC**
Lib-SVM	60.7%	41.9%	0.203
ProMate	9.9%	28.1%	0.007
PINUP	21.2%	39.5%	0.096
PPISP	27.7%	44.2%	0.146

This table shows the prediction results of different method on the test set. The first column is the method used for site prediction. The second, third, and fourth columns are average sensitivity, specificity, and MCC over all proteins in the test set. All the statistics are perform according to the classification method which classifies residues into interface and non-interface classes.

### Prediction test on the Benchmark of unbound complexes

To further verify the prediction ability of our best SVM model, prediction tests were performed on the protein-protein interaction benchmark v2.0 [40]. The unbound protein complexes in the benchmark have been divided into receptor and ligand already. Some of the receptors and ligands were discarded because they contained more than one chains. Altogether, there were 101 unbound proteins left (see Additional file 6 for the list of the 101 unbound proteins) and their interaction sites were predicted. The average sensitivity, specificity, and MCC of the prediction results of the 101 proteins were 57.5%, 32.5%, and 0.168, respectively using the SVM with core cut-off = 0.2 (Table 7).

**Table 7:** Prediction result on unbound proteins of the Benchmark

	**Sensitivity**	**Specificity**	**MCC**
Benchmark unbound	57.5%	32.5%	0.168

This table shows the prediction result on 101 unbound proteins of the Benchmark.

## Discussion

In our method, we applied the concept of core interface residue to perform the prediction. The concept of core interface residue was first purposed by Lo Conte et al. [8] and Chakrabarti et al [11]. In their paper, atoms on the interface are divided into two parts according to the buried level after binding. Their idea was adapted into our method. Those most buried residues by the interface were defined as the core interface residues. We assumed that the property around the binding site area changes gradually from the core interface to the non-interface. Therefore, the rim interface is an intermediate region between the core interface and the non-interface, which has the mixed characteristics of both.

The ratio of interface neighbours is designed to give a quantitative measure to evaluate whether a residue belongs to the core interface or the non-interface. If the ratio equals to 1, the residue is surrounded by interface residues and is an ideal core interface residue. If the ratio equal to 0, the residue has nothing to do with the interface and belong to non-interface. We can use different ratios (core cut-offs) to study different subsets of residues on the interface. In our current work, two cut-offs were used. The core interface residues using cut-off = 0.8 reflect the characteristics of residues on the centre of the interface. Their numbers are small but they have unique properties and can be better discriminated from other residues. However, the small proportion of these residues in the whole surface residues makes the prediction specificity stay at a low level. Therefore, a looser standard was used instead with the cut-off = 0.2. This leads to relative higher prediction specificity and more applicability.

The basis of interaction binding site prediction is that there are significant differences in the characteristics between the interface and non-interface residues. Sequence conservation is generally considered very important for the discrimination between the interface and non-interface residues. We also found it to be one of the most important descriptors in our method. Relative accessible surface area is also used in several site prediction methods [19,29], and proved to be effective. Our results are consistent with their results. Among many new descriptors that we tried in our binding site prediction, the side-chain environment is another important descriptor that is able to discriminate the core interface residues from the rim interface and non-interface residues. The significant effect of the side-chain environment is because it combines two properties, namely the solvent accessibility of the central residue and the degree of polarity in the exposed part of the side-chain, and provides the precise description of the characteristics of the centre residue.

The prediction results of the benchmark unbound complexes were worse than that of the test set of 50 proteins. The proteins in the benchmark are in unbound state. The proteins in the test set are in bound state. The main reason for the deterioration on the benchmark is because of the errors that come from the conformational change on the interface region of the unbound structure. An unbound training set may be a possible way to increase the general accuracy of prediction on unbound structures.

## Conclusion

In this paper, we purposed a SVM-based protein-protein interaction-site prediction method using the concept of core interface residue. We tested our method on the test set and the protein-protein interaction benchmark V2.0, and obtained reasonable prediction results for both bound and unbound structures. Through the comparison of prediction results of the test set, we showed that our method outperformed three other binding-site prediction methods. Therefore, our method shows adequate prediction ability and provides a basis for further development.

## Methods

### Generation of the data set of complex structures

The data set of complex structures consisted of 977 binary complexes. Each complex has a protein chain and an interaction partner. These complexes were extracted from PDB database [39] according to the following criterions. First, each protein chain must form a heterodimer with its interaction partner chain. Second, all the protein chains must be derived from PDB entries whose structures were solved by the X-ray diffraction method and their resolutions must be better than 3.0Å. Third, if a protein chain had contacts with more than one chains, the interaction partner chain selected was the chain that has the largest interface area. Fourth, all protein chains must be longer than 40 residues while their respective interaction partner chains could be of any length. 7610 protein chains and their respective interaction partner chains were obtained after filtering the PDB database with the above mentioned criterions. Then these protein chains were clustered according to their sequence homology by the BLASTCLUST program in the NCBI BLAST2.0 package. If the sequence alignment of any two protein chains had more than 30% identity while their alignment covered 90% of the two sequences, they would be considered to be in the same homology cluster. According to this criterion, 7610 protein chains were clustered into 977 clusters. After the sequence homology clustering, only one protein chain was kept for each cluster. Several factors were compared to decide which chain in the cluster was to be kept. First, the chain with the best resolution was kept. Second, if more than one chain was left after the comparison of resolution, then the sequence length was compared and the longest one was kept. Finally, if there was still more than one chain left, the deposition date was compared and the newest one was kept. After the three-step comparison was finished, 977 proteins were kept and they formed the data set of complexes.

### Classification of residues

The residues are divided into four classes: interior residues, core interface residues, rim interface residues, and non-interface residues.

To perform the classification, all residues of the protein chain were first divided into surface residue and interior residue. The REMOVESURFATOM program in SOFTDOCK package [42] was used to define the surface residues of a protein structure. The main-chain atoms that had no more than 22 neighbour atoms or the side-chain atoms that have no more than 16 neighbour atoms were defined as surface atoms. Two atoms were defined as neighbours to each other if their distance was less 5Å. Residues that contained surface atoms were defined as surface residues. Residues that were not defined as the surface residues were defined as interior residues. Then interface residues were picked out from the surface residues. The interface residues were defined as the surface residues that contacted with any residue on the interaction partner. A residue-residue contact was defined when the shortest distance between any pair of atoms from two residues was less than 5Å. The surface residues that did not belong to the interface residues were defined as non-interface residues. The prediction results of ProMate, PINUP, and PPISP are analyzed following the definition of interface and non-interface residues.

The classification of core interface residues and rim interface were performed according to the ratio of interface neighbours. For each surface residue, the ratio of interface neighbours is calculated as the proportion of the number of interface neighbours against the number of all its neighbours. Then we set up a cut-off for the ratio (core cut-off). The core interface residues are defined as surface residues whose ratio is no less than the cut-off. The rim interface residues are defined as surface residues whose ratio is less than the cut-off and larger than zero. The non-interface residues are defined as surface residues whose ratio is zero. Different cut-offs of ratio can give out different classification of the core and the rim interface residues. Therefore we can apply different cut-offs of the ratio to carry out the analysis of residue characteristics and build SVM models. (See Additional file 7 for detail information.)

### Construction of the residue neighbour profile

We used a residue neighbour profile to describe the local characteristics for each central or interaction residue. The residue neighbour profile consists of three parts: the central residue, the sequence neighbour residues of the central residue, and the spatial neighbour residues of the central residue.

The sequence neighbour residues contained all the residues within a window size M of the sequence centred at the central residue. The spatial neighbour residues were the N spatial nearest residues of the central residue. When searching for spatial neighbour residues, the sequence neighbour residues were excluded from consideration.

### Descriptors for amino acid residue

Each amino acid residue in the residue neighbour profile was characterized by eight descriptors including physicochemical characteristics, hydrophobic index, relative accessible surface area, secondary structure, sequence conservation, side-chain environment, sequence distance, and spatial distance.

1. Physicochemical characteristics. Physicochemical characteristics of an amino acid residue (Table 8) were described by three values: number of atoms, number of electrostatic charge, and number of potential hydrogen bonds. These values were only related to the type of amino acid and did not contain any structural information from the amino acid residue.

**Table 8:** Physiochemical characteristics of amino acids

**Amino Acid Name**	**Physicochemical characteristics**	**Amino Acid Name**	**Physicochemical characteristics**
A	(5 0 2)	M	(8 0 2)
C	(6 0 2)	N	(8 0 4)
D	(8 -1 4)	P	(7 0 2)
E	(9 -1 4)	Q	(9 0 4)
F	(11 0 2)	R	(11 1 4)
G	(4 0 2)	S	(6 0 4)
H	(10 0 4)	T	(7 0 4)
I	(8 0 2)	V	(7 0 2)
K	(9 1 2)	W	(14 0 3)
L	(8 0 2)	Y	(12 0 3)

This table shows the physiochemical characteristics value for different amino acid. The first and the third columns are one letter abbreviation of amino acids. The second and the fourth columns are the value of physiochemical characteristics for amino acids. The three numbers in the brackets are the number of atoms, the number of electrostatic charge, and the number of potential hydrogen bond.

**Figure 5:**
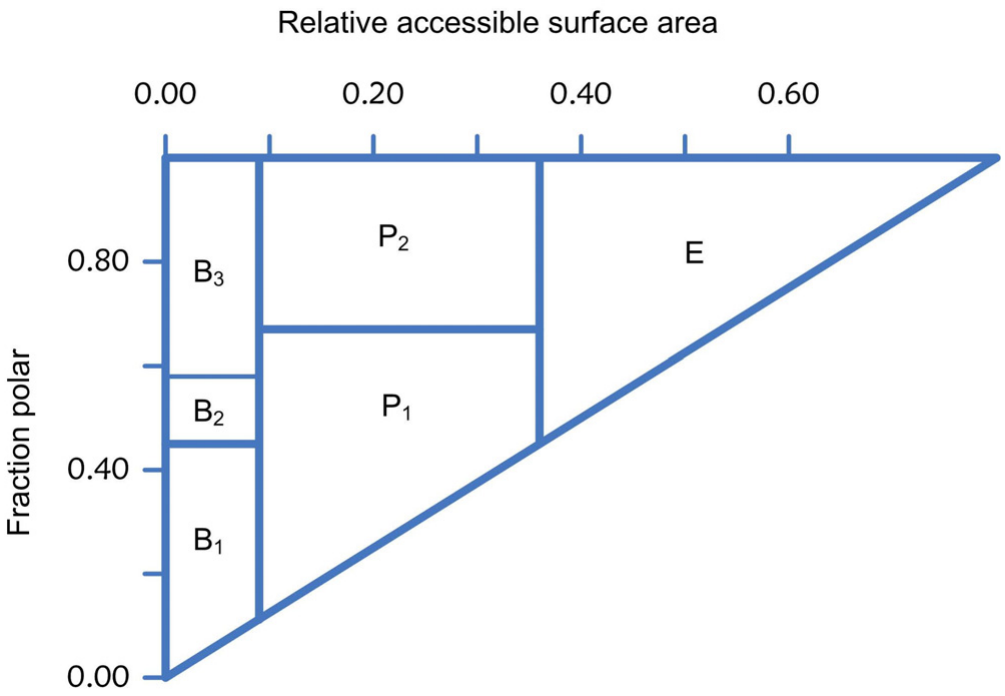
**The definition of the six local environment classes**. This figure shows the classification method of side-chain environment. RASA stands for the relative accessible surface area and FP stands for the fraction of surface area of polar atoms in the surface area of the whole side-chain. If RASA ≥ 0.36, the residue will be divided into class E (exposed). If 0.09 ≤ RASA < 0.36, the residue will be divided into class P (partial buried). Within class P, if FP < 0.67, the residue will be class P_1_, and if FP ≥ 0.67, the residue will be class P_2_. If RASA < 0.09, the residue will be divided into class B (buried). In class B, if FP < 0.45, the residue will be class B_1_, if 0.45 ≤ FP < 0.58, the residue will be class B_2_, and if FP ≥ 0.58, the residue will be class B_3_.

2. Hydrophobicity. The hydrophobicity of an amino acid residue was described by the hydrophobic index designed by Eisenberg et al. [43].

3. Relative accessible surface area. The relative accessible surface area was calculated by dividing the accessible surface area with the accessible surface area of fully-exposed amino acid. The accessible surface area of an amino acid was calculated by DSSP program [44]. The accessible surface areas of the fully exposed amino acids were according to Rost et al. [45].

4. Secondary structure. The secondary structure of an amino acid residue was also calculated by DSSP. The secondary structure was divided into three states: helix, sheet and coil. DSSP secondary structure type I, G and H were considered as helix; type E and B were considered as sheet; type T, S and blank were considered as coil.

5. Conservation score. The values of sequence conservation for amino acids were obtained by PSI-BLAST search [46] of the protein chain sequence in the Uniprot database [47]. The round of iteration was set to 3. The result of the PSI-BLAST search was a position-specific scoring matrix. The diagonal value of each residue was extracted as the value of sequence conservation.

6. Side-chain environment. Side-chain environment was first purposed by Eisenberg et al. [48] and used in his 3D-profile structural prediction method. We followed their method and divided the side-chain environment of a residue into six classes (Figure 5) according to its burial degree and the fraction of side-chain area covered by polar atoms. The details of the classification for the side-chain environment were described in Eisenberg et al. [49].

7. Sequence distance. The sequence distance was the difference of sequence numbers between a residue in the profile and the central residue.

8. Spatial distance. The spatial distance was the minimum distance between the residue in the profile and the central residue.

9. Descriptors for the central residue only. The residue and atom compositions of all sequence and space neighbours of the central residue are calculated. Atoms of amino acids are divided into 18 classes according to the work of Zhang et al. [41].

For each central residue, a residue neighbour profile of 1+M+N residues was defined and calculated. Each residue in the profile was described by its descriptors which was 48 for the central residue and 10 for the other residues in the profile. Therefore, the feature of each central residue was described by a 48+10M+10N dimensional vector, with M = 8 and N = 4 for our final SVM model.

### SVM training

977 proteins were randomly divided into a training set and a test set. 927 proteins were assigned to the training set (see Additional file 4) and 50 randomly selected proteins to the test set (see Additional file 5).

The SVM training and prediction were performed by the lib-SVM package [49]. A balanced training set comprised of equal number of core interface residues and non-core interface residues was constructed and used to carry out SVM training. Different combinations of window size M for sequence neighbour and number N for spatial neighbour were tested by the training set using 0.2 as the core cut-off. The value of CVA was used to determine which parameter set was better. The best parameters for windows size M and number N were set to be 8 and 4, respectively. The radial kernel function was used. The parameter c and gamma of the radial kernel function were optimized and set to be 1 and 1/168 respectively. When the training of SVM model was finished, the SVM model was kept for the SVM prediction.

### SVM prediction

The SVM model obtained from the training process was used to predict the interaction binding site on the protein surface. We utilized the probability estimation function of lib-SVM which can evaluate for each residue the probability to be a core interface residue.

### Evaluation of prediction results

When the optimization of parameters and the descriptor selection were performed, the cross validation accuracy (CVA) was used. The CVA was calculated by lib-SVM when performing the cross validation tests. In this paper, all CVA were calculated for the 5-fold cross validation test.

The prediction results were evaluated by sensitivity, specificity, and Matthews correlation coefficient (MCC):

$$\begin{array}{l} Sensitivity=\frac{TP}{TP+FN}\\ Specificity=\frac{TP}{TP+FP}\\ MCC=\frac{TP\times TN-FP\times FN}{\sqrt{\left(TP+FP)\times (TP+FN)\times (TN+FP)\times (TN+FN\right)}} \end{array}$$

In above equations, TP, FN, FP, and TN are true positive, false negative, false positive and true negative, respectively. Sensitivity is the fraction of the number of true positive over the number of true positive plus false negative. Specificity is the fraction of the number of true positive over the number of true positive plus false positive. The value of MCC is between 1 and -1 and higher MCC corresponds to better prediction performance.

### Drawing ROC curves and the calculation of AUC

The data of the test set is first merged into the data of core interface and the data of other residues. Then SVM model was used to make predictions on the merged test set data. The receiver operating characteristics (ROC) curves are drawn by changing the probability value cut-off output by lib-SVM. The AUC is calculated by the trapezoidal rule [50].

### Calculation of p-value

The values of residue and atom composition of the core interface, the rim interface, and the non-interface residues are submitted to a statistical test. The Welch two sample t-test in R package [51] was used to calculate the p-value. We used 0.05 as the cut-off of the probability to judge whether the difference of average between the two samples is significant.

### Program availability

The source code of our site prediction method is mainly written by PERL and C. The SVM part of our method used lib-SVM [49]. Currently, the source code of the programs can be downloaded from web-site .

## Abbreviations

CVA: cross validation accuracy; MCC: Matthews correlation coefficient; SVM: support vector machine; ROC: receiver operating characteristics; AUC: area under curve.

## Authors' contributions

LN participated in its design, wrote the code, performed the calculations, analyzed the data, and drafted the manuscript. SZ performed the calculations and analyzed the data. FJ conceived of the study, participated in its design and coordination, and drafted the manuscript. All the authors read and approved the final manuscript.

## Supplementary Material

Additional file 1 **The p-value of residue composition**. The p-value of Welch t-test for residue composition value between core interface and rim interface, between core interface and non-interface, and between rim interface and non interface. Click here for file

Additional file 2 The list of 18 atom types. Click here for file

Additional file 3 **The p-value of atom composition**. The p-value of Welch t-test for some of the atom composition value between core interface and rim interface, between core interface and non-interface, and between rim interface and non interface. Click here for file

Additional file 4 **The chain list for the training set**. List the PDB chains used in the training process. Click here for file

Additional file 5 **The chain list for the test set**. List the PDB chains in the test set. Click here for file

Additional file 6 **The chain list for the unbound proteins**. List the PDB chains of the 101 unbound proteins. Click here for file

Additional file 7 **The classification list of residues for each protein**. The classification list for each protein. After the file is decompressed, there are two directories: surfaceClass_ratio_0.2 and surfaceClass_ratio_0.8 which stands for the classification using core cut-offs 0.2 and 0.8 respectively. The *.class files in each directory contain the information of which type a residue belongs to. The first column of *.class file is the residue number in the PDB file. The second column of *.class file is the residue name. The third column of *.class file is the surface class type: 1-interior, 2-rim interface, 3-core interface, and 4-non-interface. Click here for file

## References

[B1] van Dijk ADJ, Boelens R, Bonvin AMJJ (2005). Data-driven docking for the study of biomolecular complexes. FEBS Journal.

[B2] Krogan NJ, Cagney G, Yu H, Zhong G, Guo X, Ignatchenko A, Li J, Pu S, Datta N, Tikuisis AP, Punna T, Peregrin-Alvarez JM, Shales M, Zhang X, Davery M, Robinson MD, Paccanaro A, Bray JE, Sheung A, Beattie B, Richards DP, Canadien V, Lalev A, Mena F, Wong P, Starostine A, Canete MM, Vlasblom J, Wu S, Orsi C, Collins SR, Chandran S, Haw R, Rilstone JJ, Gandi K, Thompson NJ, Musso SR, Onge PS, Ghanny S, Lam MH, Butland G, Altaf-UI AM, Kanaya S, Shilatifard A, O'Shea Weissman JS, Ingles CJ, Heghes TR, Parkinson J, Gerstein M, Wodak SJ, Emili A, Greenblatt JF (2006). Global landscape of protein complexes in the yeast Saccharomyces cerevisiae. Nature.

[B3] Phizicky E, Bastiaens PI, Zhu H, Snyder M, Fields S (2003). Protein analysis on a proteomic scale. Nature.

[B4] Smith JR, Sternberg MJ (2002). Prediction of protein-protein interactions by docking methods. Curr Opin Struct Biol.

[B5] Lensink MF, Mendez R, Wodak SJ (2007). Docking and scoring protein complexes: CAPRI 3rd edition. Proteins.

[B6] Smith GR, Fitzjohn PW, Page CS, Bates PA (2005). Incorporation of flexibility into rigid-body docking: applications in rounds 3–5 of CAPRI. Proteins.

[B7] Qin S, Zhou HX (2007). A holistic approach to protein docking. Proteins.

[B8] Lo Conte L, Chothia C, Janin J (1999). The atomic structure of protein-protein recognition sites. J Mol Biol.

[B9] Neuvirth H, Raz R, Schreiber G (2004). ProMate: a structure based prediction program to identify the location of protein-protein binding sites. J Mol Biol.

[B10] Zhou HX, Shan Y (2001). Prediction of protein interaction sites from sequence profile and residue neighbour list. Proteins.

[B11] Chakrabarti P, Janin J (2002). Dissecting protein-protein recognition sites. Proteins.

[B12] Jones S, Thornton JM (1996). Principles of protein-protein interactions. Proc Natl Acad Sci USA.

[B13] Tsai CJ, Lin SL, Wolfson HJ, Nussinov R (1997). Studies of protein-protein interfaces: A statistical analysis of the hydrophobic effect. Protein Sci.

[B14] Jones S, Thornton JM (1997). Analysis of protein-protein interaction sites using surface patches. J Mol Biol.

[B15] Tjong H, Qin S, Zhou HX (2007). PI2PE: protein interface/interior prediction engine. Nucleic Acid Res.

[B16] Hu Z, Ma B, Wolfson H, Nussinov R (2000). Conservation of polar residue as hot spots at protein interfaces. Proteins.

[B17] Ma B, Elkayam T, Wolfson H, Nussinov (2003). Protein-protein interactions: Structurally conserved residues distinguish between binding sites and exposed protein surfaces. Proc Natl Acad Sci USA.

[B18] Jones S, Thornton JM (1995). Protein-protein interactions: a review of protein dimer structures. Prog Biophys Mol Biol.

[B19] Chen H, Zhou HX (2005). Prediction of interface residues in protein-protein complexes by a consensus neural network method: test against NMR data. Proteins.

[B20] Liang S, Zhang C, Liu S, Zhou Y (2006). Protein binding site prediction using an empirical scoring function. Nucleic Acids Res.

[B21] Lichtarge O, Bourne HR, Cohen FE (1996). An evolutionary trace method defines binding surfaces common to protein families. J Mol Biol.

[B22] Lichtarge O, Sowa ME (2002). Evolutionary predictions of binding surfaces and interactions. Curr Opin Struct Biol.

[B23] Madabushi S, Yao H, Marsh M, Kristensen DM, Philippi A, Sowa ME, Lichtarge O (2002). Structural clusters of evolutionary trace residues are statistically significant and common in proteins. J Mol Biol.

[B24] Yao H, Kristensen DM, Mihalek I, Sowa ME, Shaw C, Kimmel M, Kavraki L, Lichtarge O (2003). An accurate, sensitive, and scalable method to identify functional sites in protein structures. J Mol Biol.

[B25] Yao H, Mihalek I, Lichtarge O (2006). Rank information: a structure-independent measure of evolutionary trace quality that improves identification of protein functional sites. Proteins.

[B26] Aloy P, Querol E, Aviles FX, Sternberg MJE (2001). Automated structure-based prediction of functional sites in proteins: applications to assessing the validity of inheriting protein function from homology in genome annotation and to protein docking. J Mol Biol.

[B27] de Vries SJ, van Dijk ADJ, Bonvin AMJJ (2006). WHISKY: What information does surface conservation yield? Application to data-driven docking. Proteins.

[B28] Chung JL, Wang W, Bourne PE (2006). Exploiting sequence and structure homologs to identify protein-protein binding sites. Proteins.

[B29] Bradford JR, Needham CJ, Bulpitt AJ, Westhead DR (2006). Insights into protein-protein interfaces using a Bayesian network prediction method. J Mol Biol.

[B30] Bradford JR, Westhead DR (2005). Improved prediction of protein-protein binding sites using a support vector machines approach. Bioinformatics.

[B31] Fariselli P, Pazos F, Valencia A, Casadio R (2002). Prediction of protein-protein interaction sites in heterocomplexes with neural networks. Eur J Biochem.

[B32] Yan C, Dobbs D, Honavar D (2004). A two-staged classifier for identification of protein-protein interface residues. Bioinformatics.

[B33] Koike A, Takagi T (2004). Prediction of protein-protein interaction sites using support vector machines. Protein Eng Des Sel.

[B34] Block P, Paern J, Hullermeier E, Sanschagrin P, Sotriffer CA, Klebe G (2006). Physicochemical descriptors to discriminate protein-protein interactions in permanent and transient complexes selected by means of machine learning algorithms. Proteins.

[B35] Armon A, Graur D, Ben-Tal N (2001). ConSurf: An algorithm tool for the identification of functional regions in proteins by surface mapping of phylogenetic information. J Mol Biol.

[B36] del Sol Mesa A, Pazos F, Valencia A (2003). Automatic methods for predicting functionally important residues. J Mol Biol.

[B37] Darnell SJ, Page D, Mitchell JC (2007). An automated decision-tree approach to predicting protein interaction hot spots. Proteins.

[B38] Kufareva I, Budagyan L, Raush E, Totrov M, Abagyan R (2007). PIER: protein interface recognition for structural proteomics. Proteins.

[B39] Berman HM, Westbrook J, Feng Z, Gilliland G, Bhat TN, Weissig H, Shindyalov IN, Bourne PE (2000). The Protein Data Bank. Nucleic Acids Res.

[B40] Mintseris J, Wiehe K, Pierce B, Anderson R, Chen R, Janin J, Weng Z (2005). Protein-Protein Docking Benchmark 2.0: an update. Proteins.

[B41] Zhang C, Vasmatzis G, Cornette JL, DeLisi C (1997). Determination of atomic desolvation energies from the structures of crystallized proteins. J Mol Biol.

[B42] Li N, Sun Z, Jiang F (2007). SOFTDOCK application to protein-protein interaction benchmark and CAPRI. Proteins.

[B43] Sweet RM, Eisenberg D (1983). Correlation of sequence hydrophobicities measures similarity in three dimensional protein structure. J Mol Biol.

[B44] Kabsch W, Sandor C (1983). Dictionary of protein secondary structure: pattern recognition of hydrogen-bonded and geometrical features. Biopolymers.

[B45] Rost B, Sander C (1994). Conservation and prediction of solvent accessibility in protein families. Proteins.

[B46] Altschul SF, Madden TL, Schaffer AA, Zhang J, Zhang Z, Miller W, Lipman DJ (1997). Gapped BLAST and PSI-BLAST: a new generation of protein database search programs. Nucleic Acids Res.

[B47] The UniProt Consortium (2008). The Universal Protein Resource (Uniprot). Nucleic Acids Res.

[B48] Bowie JU, Luthy R, Eisenberg D (1991). A method to identify protein sequences that fold into a known three-dimensional structure. Science.

[B49] Fan R, Chen P, Lin C (2005). Working set selection using the second order information for training SVM. J Mach Learn Res.

[B50] Press WH, Teukolsky SA, Vetterling WT, Flannery BP (1995). Numerical Recipes in C.

[B51] R Development Core Team (2008). R: A Language and Environment for Statistical Computing Austria.

